# A Vitiligo-like Cutaneous Reaction Induced by Ribociclib in Advanced Breast Cancer: An Unusual Case Report from Colombia

**DOI:** 10.3390/diseases13050158

**Published:** 2025-05-19

**Authors:** John Fernando Montenegro, Giovanna Patricia Rivas-Tafurt, Sinthia Vidal-Cañas, Miguel Ángel Diaz-Diaz, Cesar Eduardo Bermudez, Daniel Florez, Andres Felipe Bravo-Gustin, Yamil Liscano

**Affiliations:** 1 Grupo de Investigación en Salud Integral (GISI), Facultad de Salud, Universidad Santiago de Cali, Cali 760035, Colombia; sinthia.vidal00@usc.edu.co (S.V.-C.); miguel1diaz996@gmail.com (M.Á.D.-D.); andres311990405@gmail.com (A.F.B.-G.); 2 Grupo de Investigación en Genética, Fisiología y Metabolismo (GEFIME), Ciencias de la Salud Universidad Santiago de Cali, Cali 760035, Colombia; giovanna.rivas@gclinicadeoccidente.com; 3 Programa de Especialización en Medicina Interna, Facultad de Salud, Universidad Santiago de Cali, Cali 5183000, Colombiajkl1999@hotmail.com (D.F.); 4 Departamento de Investigación y Educación, Clínica de Occidente S.A., Cali 760046, Colombia

**Keywords:** vitiligo, ribociclib, autoimmune, hypopigmented, breast cancer

## Abstract

Background: Cutaneous toxicities associated with CDK4/6 inhibitors are uncommon but may affect treatment adherence. We present the case of a patient with advanced breast cancer who developed vitiligo-like lesions after initiating ribociclib, contributing to the growing evidence of this under-recognized adverse effect. Methods: We present the case of a 72-year-old woman diagnosed in 2007 with early-stage, luminal A, HER2-negative breast cancer, initially treated with surgery and tamoxifen. In 2022, she experienced locoregional recurrence with bone metastases. In January 2023, she began treatment with ribociclib plus letrozole. Two months later, she developed intense pruritus, xerosis, and paresthesia, followed by hypopigmented lesions on her face and upper extremities. Clinical evaluation, supported by photographs and a skin biopsy (led to a diagnosis of ribociclib-induced vitiligo. Management included dose adjustments to the ribociclib and dermatologic treatments, including topical corticosteroids, antihistamines, and short courses of oral prednisone. Results: By September 2024, her skin lesions had stabilized and her pruritus improved with a reduced dose of ribociclib (one tablet per day). However, the hypopigmented patches persisted, mainly on her face and extremities. Despite these cutaneous effects, she maintained an acceptable quality of life and continued effective oncologic treatment. Conclusions: This case highlights the importance of early recognition and management of ribociclib-related cutaneous toxicities. A multidisciplinary approach is essential to minimize adverse effects without compromising therapeutic efficacy. Further research into the dermatologic manifestations of targeted therapies is needed to optimize patient care.

## 1. Introduction

Vitiligo is an autoimmune skin disorder characterized by the progressive loss of melanocytes, leading to hypopigmented lesions that significantly impact patients’ quality of life [1]. Its estimated prevalence in the United States is 1.38% based on self-reported data and 0.76% based on medical diagnosis. The condition affects men and women equally, showing no racial or socioeconomic predilections. It can manifest at any age, though it is most common between the second and third decades of life. Approximately one-third of cases occur in children, and 70–80% of adults develop vitiligo before the age of 30 [2,3,4].

Vitiligo-like skin lesions have been reported as rare but documented adverse effects in breast cancer patients treated with CDK4/6 inhibitors such as ribociclib and palbociclib. Recent studies, including that by Sollena et al. (2021) [5], have described cases of acquired hypopigmented lesions associated with these drugs. In that series, a higher frequency was observed with ribociclib and the involvement of photoexposed areas [6].

While vitiligo is a recognized autoimmune condition, certain medications, including cancer therapies, have been associated with drug-induced vitiligo-like reactions [7]. Ribociclib is a selective inhibitor of CDK4 and CDK6 kinases, used in the treatment of breast cancer at both the early and advanced stages. Its mechanism of action involves blocking CDK4/6-mediated phosphorylation of the retinoblastoma protein, halting the cell cycle at the G1–S transition and preventing cell division [8]. Despite its effectiveness, ribociclib is associated with adverse effects such as hematological toxicity, hepatotoxicity (12%), QT interval prolongation (11%), and cutaneous toxicities, including alopecia (23%) and pruritus (15–20%) [8,9]. Rarely, drug-induced vitiligo-like disorders have been reported, although their exact incidence remains poorly documented [10]. On the other hand, a systematic review by Silvestri et al. (2021) [10], which aimed at evaluating the incidence and clinical spectrum of cutaneous adverse events in breast cancer patients treated with cyclin-dependent kinase 4/6 inhibitors, included 41 articles. It found that a multicenter study of 16 patients with stage IV breast cancer described vitiligo-like lesions observed during treatment with CDK4/6 inhibitors (14 out of 16 treated with ribociclib and 2 out of 16 with palbociclib), identifying it as a rare but well-documented manifestation [10,11,12]. However, a recent European multicenter study by Sollena et al. (2021) [5] has documented vitiligo-like lesions in patients treated with CDK4/6 inhibitors, establishing them as a recognized adverse event [5].

On the other hand, drug-induced vitiligo-like disorders have been reported only rarely, and their exact incidence remains poorly documented. In one study, twenty-one patients developed vitiligo-like lesions while receiving treatment with palbociclib and ribociclib. The onset time was slightly delayed, with a median of 9.8 months [13]. Additionally, similar cases have been observed, with ribociclib being more frequently associated with these reactions, while palbociclib showed a lower incidence [13]. Finally, we have recently described vitiligo-like reactions associated with ribociclib, with a lesser frequency of occurrence in relation to palbociclib [6,13]. Therefore, vitiligo-like skin lesions have been infrequently reported in the literature, although they are recognized in clinical practice as adverse events associated with the use of CDK4/6 inhibitors. Moreover, severe cutaneous toxicities have been documented as uncommon. Despite their low prevalence in studies, these manifestations should not be considered unknown [13].

Research into ribociclib’s cutaneous effects has provided insights into its safety profile. According to studies such as the MONALEESA trials, the incidence of ribociclib-induced cutaneous toxicities is approximately 14.3%. The most common skin reaction is eczematous dermatitis, followed by maculopapular rashes and lichenoid dermatitis. Severe cutaneous adverse events have been documented in fewer than 1% of cases, with no evidence that these toxicities affect patient prognosis. However, vitiligo associated with ribociclib and other immune checkpoint inhibitors, such as PD-1/PD-L1 inhibitors, warrants further investigation [8,9,14]. Understanding these adverse events is critical to optimizing patient care.

This report describes the case of a patient with luminal A, HER2-negative breast cancer who developed hypopigmented lesions consistent with vitiligo after initiating ribociclib therapy. This finding underscores the importance of monitoring cutaneous toxicities in oncology patients and highlights the need for further research into the interactions between antitumor treatments and autoimmune dermatological conditions. Additionally, it emphasizes the significance of an integrated clinical management approach that considers these toxicities as an essential component of therapeutic monitoring [9,15].

## 2. Case Presentation

### 2.1. Initial Diagnosis and Early Management

**Figure 1:**
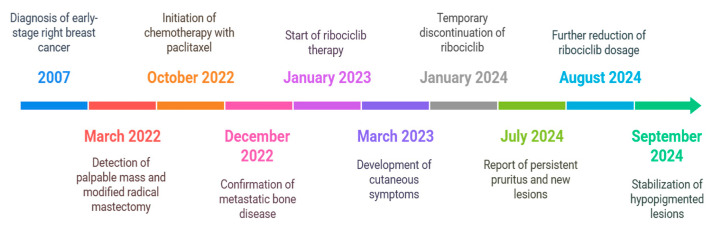
Timeline of clinical events and management of ribociclib-related skin toxicities. The timeline illustrates key clinical milestones in the diagnosis, treatment, and management of a 72-year-old female patient with breast cancer and ribociclib-induced hypopigmented lesions.

A 72-year-old female patient was diagnosed in 2007 with early-stage right breast cancer (CTxNxM1) (see Figure 1). At the time, she underwent a right quadrantectomy with sentinel lymph node biopsy and was started on adjuvant therapy with tamoxifen at a dose of 20 mg daily. This regimen was maintained for five years. No recurrences were observed until 2022.

### 2.2. Recurrence and Progression

In March 2022, the patient noticed a palpable mass in the lower quadrant of her right breast. The mass exhibited progressive growth but was not associated with nipple discharge, inversion, or skin discoloration. A modified radical mastectomy with levels I and II lymph node dissection was performed. Subsequently, in October 2022, the patient initiated chemotherapy with paclitaxel (80–100 mg/m^2^ weekly) for three cycles. In December 2022, a bone scan confirmed metastatic bone disease. The treatment was adjusted to include denosumab (120 mg subcutaneously every four weeks), letrozole (2.5 mg daily), and ribociclib (600 mg daily, three 200 mg tablets). The ribociclib therapy formally began in January 2023.

### 2.3. Development of Cutaneous Symptoms

**Figure 2:**
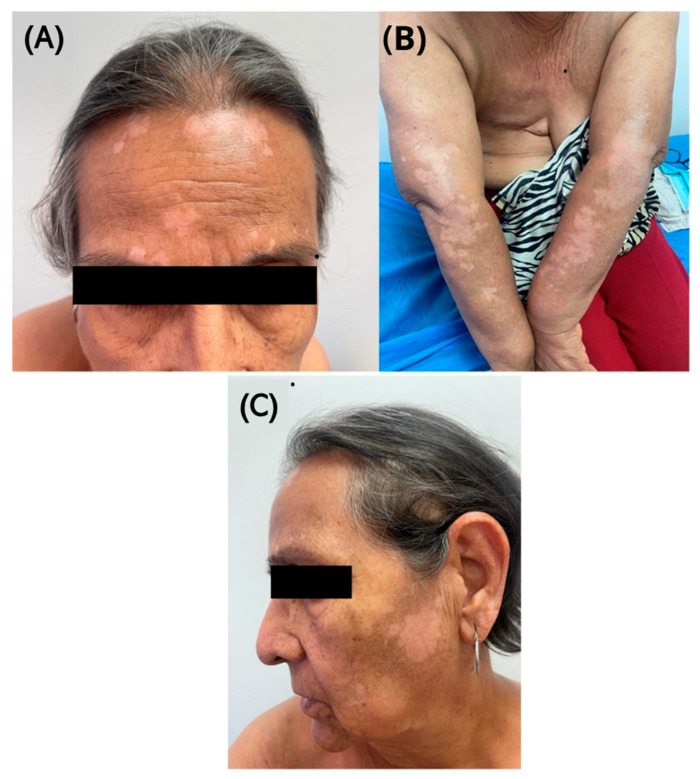
Hypopigmented lesions associated with ribociclib therapy: Clinical presentation. (**A**) Well-demarcated hypopigmented macules in the frontal and periocular regions, without signs of inflammation or scaling. The lesions display a symmetric pattern in sun-exposed areas. (**B**) Hypopigmented macules on the forearms and dorsal surfaces of the hands, symmetrically distributed. The borders are well defined, with no erythema or associated atrophy. (**C**) Hypopigmented lesions with sharp borders in the bilateral malar and preauricular regions. They predominantly affect sun-exposed areas, with the surrounding skin preserved.

By March 2023, the patient developed intense pruritus, xerosis, and paresthesia, followed by hypopigmented lesions. These symptoms persisted into June, prompting the oncology team to adjust the ribociclib dose by 15% with an intermittent regimen (three tablets one day and two tablets the next, followed by a 10-day break), while continuing letrozole. She was referred to the dermato-oncology service, where hypopigmented macules were observed on her forehead, cheeks, and forearms (Figure 2), consistent with vitiligo.

**Figure 3:**
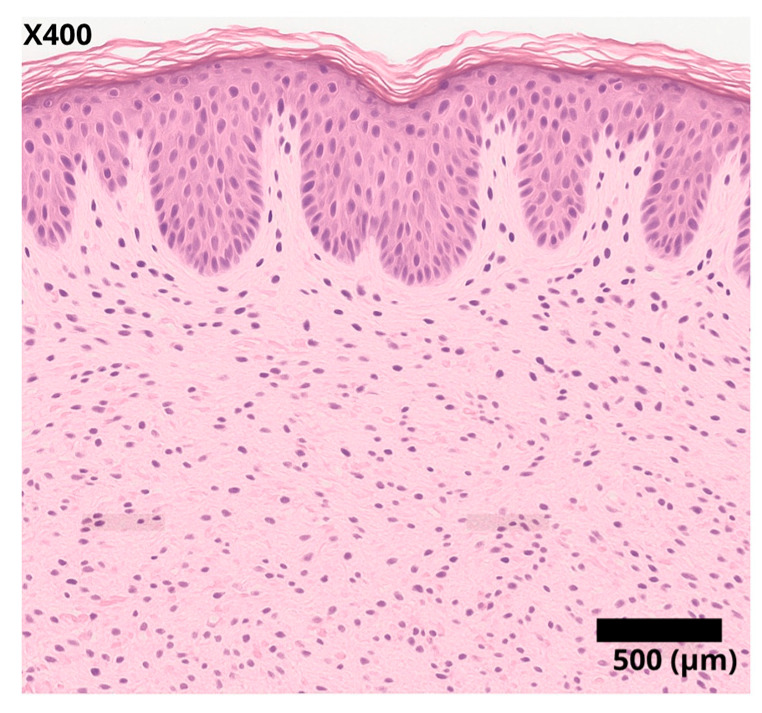
Histopathological section of skin affected by vitiligo (hematoxylin and eosin staining, original magnification ×400). The epidermis exhibits preserved stratification and architecture, but there is a notable absence or significant reduction of melanocytes in the basal layer. The superficial dermis shows a mild perivascular inflammatory infiltrate without evidence of collagen or vascular damage. These histopathological findings confirm the diagnosis of vitiligo, characterized by selective melanocyte loss and minimal inflammatory response.

Although the diagnosis was clinical, the case description was supplemented by the use of a Wood’s lamp, which notably intensified the white color of the lesion, and additionally by histopathology (Figure 3), where an inflammatory infiltrate was observed in the superficial dermis, along with a decrease in melanocytes and residual melanin. Treatment with topical kellin cream and clobetasol was initiated.

### 2.4. Further Adjustments and Monitoring

In January 2024, due to persistent pruritus and rash, ribociclib was temporarily discontinued for two weeks. The therapy was reintroduced at a 10% reduced dose (540 mg daily). The symptomatic treatment included desloratadine (5 mg daily) and acetylcysteine (600 mg twice daily), resulting in a partial improvement of the hypopigmented lesions and rash. Hydroxyzine was discontinued due to a lack of additional benefit. The ribociclib dose was further reduced to 400 mg daily (two 200 mg tablets), leading to a significant reduction in the pruritus and rash.

### 2.5. Progression and Current Management

In July 2024, the patient reported persistent intense pruritus and the appearance of new hypopigmented lesions on her face and extremities. In August, as the lesions progressed, ribociclib was further reduced to 200 mg daily (one tablet). The additional treatments included hydroxyzine (25 mg nightly), a short course of prednisone (20 mg daily), and loratadine (10 mg daily). By September 2024, the pruritus and rash improved, but the hypopigmented lesions persisted, particularly in the frontal and malar regions of the face and extremities.

Therefore, the patient continues on ribociclib at 200 mg daily and is under interdisciplinary follow-up by the oncology and dermato-oncology teams. She has shown progressive improvement in the xerosis and stabilization of the hypopigmented lesions. Management now includes 0.1% tacrolimus ointment and phototherapy, with 48 sessions completed, leading to stabilization of the achromic lesions. However, the vitiligo remains a clinical challenge.

Finally, the patient initially expressed concern about the aesthetic impact of vitiligo, but after receiving information about its benign nature, she achieved better emotional adaptation. She did not present with significant anxiety, although she expressed concern about the progression of the lesions. Interdisciplinary follow-up helped reduce her concern and improve her quality of life.

## 3. Discussion

### 3.1. Challenges in Managing HR+/HER2− Metastatic Breast Cancer

This case highlights the clinical challenges associated with managing HR+/HER2− metastatic breast cancer, particularly in patients treated with CDK4/6 inhibitors [15]. While these therapies are highly effective in delaying tumor progression and improving progression-free survival, they can also cause rare but significant adverse effects, such as vitiligo [16]. In this case, the patient developed hypopigmented lesions while receiving ribociclib, underscoring the need for multidisciplinary monitoring to address these complications without compromising the treatment efficacy. In addition, this patient underwent a comprehensive treatment regimen, including surgery, chemotherapy, and hormonal therapy, followed by CDK4/6 inhibitors and aromatase inhibitors to manage bone metastases, inhibit cell growth, and prevent future recurrences.

### 3.2. Dermatological Toxicities of CDK4/6 Inhibitors

CDK4/6 inhibitors, such as ribociclib, play a critical role in treating HR+/HER2− metastatic breast cancer by halting cell progression during the G1–S phase of the cell cycle. While vitiligo is a rare adverse effect, its occurrence, as observed in this case, highlights the need for careful monitoring of cutaneous toxicities to ensure uninterrupted treatment [8,17].

In the study by Sollena et al. (2023) [6], a retrospective analysis was conducted to investigate the prevalence, types, and management of cutaneous adverse events during treatment with CDK4/6 inhibitors. The study included 79 adult female patients with advanced breast cancer, who experienced a total of 125 dermatologic adverse events during CDK4/6 inhibitor treatment across eleven centers. The most common cutaneous reactions were pruritus (49/79 patients), alopecia (25/79), and eczematous lesions (24/79) [6].

### 3.3. Strengths of the Case Report

The strength of this report lies in its thorough evaluation, which enabled the precise and timely identification of the adverse effects and their effective management [15]. As a result, the patient experienced an improvement in her quality of life. Documenting this case complements similar reports in the literature; for example, the findings of our case align with the broader European experience documented in the EADV Task Force study (Sollena et al., 2023 [6]), which reported similar dermatological adverse events in patients receiving CDK4/6 inhibitors [6]. This is crucial for helping clinicians make informed decisions regarding the use of CDK4/6 inhibitors [16]. These decisions require careful risk–benefit analysis.

### 3.4. Review of Similar Cases in the Literature

Several cases in the literature highlight the challenges of managing ribociclib-associated cutaneous adverse events (see Table 1). Sharaf et al. (2022) [13] described a 71-year-old woman who developed vitiligo after 20 days of ribociclib and aromatase inhibitor treatment, with persistent depigmentation despite corticosteroids [9]. Pasqualoni et al. (2023) [9] reported two cases: a 46-year-old with severe pruritus and hypopigmented lesions partially relieved by dose reduction and topical immunosuppressants, and an 80-year-old who developed vesicular rashes and permanent hypopigmented lesions leading to CDK4/6 discontinuation despite tumor stability. Türkel et al. (2023) [14] described vitiligo-like lesions in a 56-year-old woman on ribociclib, which were resistant to corticosteroid treatment. Borroni et al. (2024) [8] analyzed multiple cases, proposing severity-based management strategies, including dose adjustments, antihistamines, emollients, and corticosteroids, alongside a classification system guiding CDK4/6 dose reductions. [8].

The management of cutaneous toxicities associated with CDK4/6 inhibitors is an evolving field, with growing evidence derived from cohort studies. In the present case, a dose reduction and topical treatment were chosen, resulting in a favorable response. However, in the event of lesion progression or deterioration in the patient’s quality of life, it is essential to consider alternative strategies.

Several cases in the literature highlight the challenges when managing ribociclib-associated cutaneous adverse events. One case described a 70-year-old woman with HR-positive, HER2-negative metastatic breast cancer who was treated for 8 months with letrozole and ribociclib. After six weeks, she developed asymptomatic hypopigmented macules on the face and neck. Wood lamp examination revealed bright white, sharply demarcated lesions, confirming the diagnosis of vitiligo. This is a rare adverse event associated with CDK4/6 inhibitors, possibly related to premature melanocyte apoptosis. Management was challenging, using topical immunosuppressants and oral corticosteroids, with a partial response [18].

When managing cutaneous toxicities associated with CDK4/6 inhibitors, it is important to consider alternative therapeutic options if these reactions worsen or affect the patient’s quality of life. In cases of significant skin toxicity that does not respond to standard management strategies, switching to a different CDK4/6 inhibitor or adjusting the treatment regimen may offer a solution. This decision requires careful consideration of the patient’s tumor response, the severity of the adverse event, and the potential benefits and risks associated with alternative inhibitors.

One such alternative is demonstrated in a retrospective cohort study involving 324 patients with advanced breast cancer treated with palbociclib, which reported that 14.2% experienced cutaneous toxicities, with maculopapular rash and xerosis being the most common. Although most cases were grade 1–2, 10% required temporary discontinuation and dose adjustment, while two patients were switched to abemaciclib, resulting in complete resolution of symptoms [19].

A study included 79 patients with advanced breast cancer treated with CDK4/6 inhibitors, documenting a total of 165 cutaneous adverse events. The most frequent reactions were pruritus (62%), alopecia (32%), and eczematous lesions (31%). Most skin toxicities were mild (grade 1–2) and occurred after a median of 6.5 months of treatment. Only 5% of patients required treatment discontinuation due to the severity of the skin lesions. Most reactions were managed with topical treatments, such as high-potency corticosteroids, and in some cases, UVA/UVB phototherapy was used. It is worth noting that the majority of patients in this study received palbociclib (64.6%), followed by ribociclib (21.5%) and abemaciclib (13.9%), allowing for a more direct comparison with the case presented. These findings reinforce the notion that, although cutaneous toxicities associated with CDK4/6 inhibitors are common, they are generally manageable and rarely require permanent treatment discontinuation [6].

Future studies should focus on identifying predictive biomarkers of cutaneous toxicity that would allow for the selection of the most appropriate CDK4/6 inhibitor from the beginning of treatment. In the meantime, clinical decisions should be personalized, based on the severity of the toxicity, its impact on quality of life, the tumor response, and the availability of alternative therapeutic options.

### 3.5. Multidisciplinary Management Approach

Anders et al. (2020) emphasized the significance of targeted therapies in breast oncology, noting that while CDK4/6 inhibitors are effective for HR+/HER2- breast cancer, they may cause adverse effects affecting multiple systems, including the dermatological events observed in this case [17]. This underscores the importance of a multidisciplinary approach, integrating oncologists and dermatologists to balance treatment efficacy with quality of life [20].

Morikawa et al. (2016) [21] suggested that vitiligo development in patients receiving immune checkpoint inhibitors may be linked to better survival, although this remains an unconfirmed observation [22].

This has been compared with other European studies, such as that by Sollena et al. (2023) [6], which concluded that most cases occur in breast cancer patients treated with any CDK4/6 inhibitor. They recommended continuing treatment despite cutaneous reactions in the majority of patients exposed to dermatological toxicities. However, it remains a therapeutic challenge. Other European studies highlighted that vitiligo-like skin lesions may appear at the later stages of ribociclib treatment [13]. The use of medium- to high-potency topical corticosteroids should be considered as the treatment of choice [6]

Additionally, Bang et al. (2024) conducted a multicenter retrospective study reviewing the vitiligo-like lesions in breast cancer patients treated with CDK4/6 inhibitors. It analyzed 10 cases from academic centers in the United States and Europe, describing the clinical characteristics, progression, and management. The study highlighted the persistence of the lesions despite treatments and investigated ruxolitinib as a potential option. The findings provided valuable insights into the dermatologic adverse events in oncology [23].

### 3.6. Limitations and Future Research

This report highlights a key limitation: the lack of prospective studies systematically evaluating cutaneous adverse events associated with ribociclib. Future research should explore the underlying immunological mechanisms and develop clear guidelines for managing these toxicities Addressing these gaps is essential for ensuring comprehensive and personalized care for metastatic breast cancer patients [5].

There is a risk of underreporting mild cutaneous events that were not documented during oncology consultations. Standardized dermatological evaluations were not performed for all the patients. Finally, the long-term follow-up regarding the resolution of toxicities was unclear in most patients [19].

## 4. Conclusions

Targeted therapies have revolutionized the treatment of breast cancer but present new challenges, including dermatological toxicities such as ribociclib-associated vitiligo. Effective management of these complications requires a multidisciplinary and personalized approach to maximize therapeutic benefits while minimizing adverse effects. This case underscores the importance of timely identification and tailored management of rare adverse events, emphasizing the need to balance the clinical manifestations, the severity, and the risks and benefits of treatment. Experiences from such cases enhance our understanding of complex conditions and provide valuable insights for developing individualized therapeutic strategies.

Although these adverse reactions are not life-threatening, they can significantly impact the patient’s quality of life. With thorough monitoring and personalized interventions, including topical or systemic treatments, these toxicities can be effectively managed, offering a framework for addressing rare side effects during cancer therapy. CDK4/6 inhibitor therapies used in the treatment of breast cancer have significantly improved therapeutic outcomes. However, this approach requires personalized and multidisciplinary attention due to its potential impact on patients. As a future recommendation, it is suggested to study the different types of cutaneous involvement associated with this treatment. Additionally, investigating the predictive biomarkers and biomolecular reactions that may lead to these conditions is proposed, aiming to provide more personalized treatment based on a risk–benefit analysis for breast cancer patients.

## Figures and Tables

**Table 1:** Comparison of cases and outcomes.

Author	Age and Gender	Clinical Background	Clinical Manifestation	Drug Administered	Treatment
Sharaf et al. (2022) [13]	71, female	HR+ HER2− breast cancer	Vitiligo, pruritus, alopecia	Aromatase inhibitor, ribociclib	Topical corticosteroids
Pasqualoni et al. (2023) [9]	46, female	HR+ HER2− breast cancer	Erythematous lesions, hypopigmentation, pruritus	Aromatase inhibitor, ribociclib	Dose reduction, short steroid course
Türkel et al. (2023) [14]	56, female	HR+ HER2− breast cancer with metastases	Vitiligo-like hypopigmentation	Aromatase inhibitor, ribociclib	Continued ribociclib, topical/oral corticosteroids
Pasqualoni et al. (2023) [9]	80, female	HR+ HER2− breast cancer	Vesicular rashes, persistent hypopigmentation	Aromatase inhibitor, ribociclib	Therapy discontinuation
Nicolás Silvestre Torner et al. (España, 2022) [18]	70, woman	Hormone receptor-positive, HER2-negative	Asymptomatic hypopigmented macules on face and neck; Wood lamp: bright white sharply delineated lesions	Ribociclib + letrozole	Dermatology referral; topical immunosuppressants + oral corticosteroids (partial response); avoid systemic immunosuppressants
Sumir Chawla et al. (2021) [19]	70 (average age)	Advanced breast cancer (324 patients in total)	Maculopapular rash, xerosis, pruritus, alopecia, eczematous lesions	Palbociclib	Topical treatments (corticosteroids), UVA/UVB phototherapy, dose adjustments, temporary discontinuation in some cases
Sollena P et al. (2023) [6]	Not specified	Cancer patients treated with CDK4/6 inhibitors	Pruritus, alopecia, erythema, acneiform eruptions, eczematous lesions	CDK4/6 inhibitors (various)	Topical corticosteroids, antihistamines, dose reduction, temporary discontinuation, dermatology referral

## Data Availability

Data are contained within the article.
